# Cryo-ET suggests tubulin chaperones form a subset of microtubule lumenal particles with a role in maintaining neuronal microtubules

**DOI:** 10.1073/pnas.2404017121

**Published:** 2025-01-31

**Authors:** Saikat Chakraborty, Antonio Martinez-Sanchez, Florian Beck, Mauricio Toro-Nahuelpan, In-Young Hwang, Kyung-Min Noh, Wolfgang Baumeister, Julia Mahamid

**Affiliations:** ^a^Department of Molecular Structural Biology, Max Planck Institute of Biochemistry, Martinsried 82152, Germany; ^b^Institute of Neuropathology and Cluster of Excellence “Multiscale Bioimaging: From Molecular Machines to Networks of Excitable Cells”, University Medical Center Göttingen, Göttingen 37075, Germany; ^c^Research group CryoEM Technology, Max Planck Institute of Biochemistry, Martinsried 82152, Germany; ^d^Structural and Computational Biology Unit, European Molecular Biology Laboratory, Heidelberg 69117, Germany; ^e^Genome Biology Unit, European Molecular Biology Laboratory, Heidelberg 69117, Germany; ^f^Cell Biology and Biophysics Unit, European Molecular Biology Laboratory, Heidelberg 69117, Germany

**Keywords:** primary neurons, hiPSC-derived neurons, microtubule lattice damage, in situ cryoelectron tomography, subtomogram averaging

## Abstract

The functional architecture of the long-lived neuronal microtubule (MT) cytoskeleton is maintained by various MT-associated proteins (MAPs), most of which are known to bind to the MT outer surface. However, electron microscopy (EM) has long ago revealed the presence of particles inside the lumens of neuronal MTs, of yet unknown identity and function. Here, we use cryogenic electron tomography (cryo-ET) to analyze the three-dimensional (3D) organization and structures of MT lumenal particles in primary hippocampal neurons, human induced pluripotent stem cell–derived neurons, and pluripotent and differentiated P19 cells. We obtain in situ density maps of several lumenal particles from the respective cells and detect common structural features underscoring their potential overarching functions. Mass spectrometry-based proteomics combined with structural modeling suggest that a subset of lumenal particles could be tubulin-binding cofactors (TBCs) bound to tubulin monomers. A different subset of smaller particles, which remains unidentified, exhibits densities that bridge across the MT protofilaments. We show that increased lumenal particle concentration within MTs is concomitant with neuronal differentiation and correlates with higher MT curvatures. Enrichment of lumenal particles around MT lattice defects and at freshly polymerized MT open-ends suggests a MT protective role. Together with the identified structural resemblance of a subset of particles to TBCs, these results hint at a role in local tubulin proteostasis for the maintenance of long-lived neuronal MTs.

## Significance

Microtubules (MTs) form the basis of the polarized cytoskeleton in neurons, and their structural integrity is essential to many neuronal functions. Stabilization of MTs has so far been attributed to proteins that predominantly bind on the MT outer surface. Yet, the identity and role of abundant particulate material seen inside the MT lumen remained unknown for decades. Here, we reveal the three-dimensional organization of MT lumenal particles and pinpoint their common morphological features in various neuronal cells. Our analyses indicate that these common components could be tubulin-binding cofactors that are essential for tubulin biogenesis in all cell types. Our data thus suggest a new functional role for MT lumenal particles in maintaining neuronal MT structural integrity.

The microtubule (MT) cytoskeleton plays an essential role in neuronal morphogenesis (1), supporting axonal trafficking (2), signal transduction (3), axon guidance (4), and synapse formation (5). Irregularities in MTs lead to abnormal morphogenesis and ultimately to detrimental neurodevelopmental defects (6–8). Thus, the neuronal MT cytoskeleton is maintained by complex layers of regulatory mechanisms that include a pool of neuron-specific MT-associated proteins (MAPs) (9). These MAPs predominantly bind to the outer surface of the hollow MT lattice and regulate the dynamics as well as material properties of the MT cytoskeleton (10–12). Early conventional EM studies further showed the presence of periodically arranged particles within the MT lumen of insect epithelia (13), spermatids (14), and blood platelets (15). Cryoelectron tomography (cryo-ET) studies have since unambiguously confirmed the presence of such MT lumenal particles in unstained, frozen-hydrated sections of different cells, and showed their high abundance particularly in neurons (16–25), but not inside MTs polymerized in vitro from purified brain tubulin (17). These studies suggest that the in vivo repertoire of neuronal MAPs could be more complex than perceived; to date, our knowledge about the structures, molecular identities, or functions of neuronal MT lumenal particles remains limited.

The MT lumen, with a diameter of approximately 17 nm, is largely secluded from the cytoplasm except for the two filament ends and lateral openings that form upon regulated severing and/or structural defects (26, 27). Nevetheless, regulation of MT stability by selective localization of MAPs or posttranslational modification (PTM) in the MT lumen may provide a favorable mechanism, since these processes should not interfere with the multitude of trafficking events happening on the MT outer surface (28). Such a mechanism is indeed supported by findings of MT inner-proteins (MIPs) in extraordinary stable cortical MTs in parasites and specialized doublet MTs in cellular appendages such as cilia and flagella that maintain the structural integrity of the MT lattice during extreme mechanical deformations caused during propulsive motion (29–33). However, the roles of MIPs in cytoplasmic MTs remain poorly described. Exceptions are studies showing acetylation of lumenal K-40 of α-tubulin by tubulin acetyltransferase (TAT) that increases MT mechanical resilience, lattice integrity, and longevity (34–36). Not surprisingly, acetylated MTs are an integral part of axonal MT bundles that function as highways for intracellular transport (37). Therefore, TATs have been suggested to be a component of the lumenal particles (38), as well as deacetylases such as HDAC6 that access the MT lumen by hitchhiking on the MT plus-end tracking protein EB1 for removing lumenal acetylation marks (39, 40). A recent report suggests that MAP6 could also reside in the MT lumen, and perform similar functions as MIPs in MT doublets by stabilizing a coiled architecture of neuronal MTs (41). However, there is no direct structural evidence to show that these proteins are components of MT lumenal particles in neurons. Thus, major questions remain as to the identities and functions that such abundant particles perform in the neuronal MT cytoskeleton.

In this study, we utilized in situ cryo-ET to elucidate the nanometer-scale organization and molecular morphologies of lumenal particles in three vitrified neuronal cell types: rodent primary hippocampal neurons, human induced pluripotent stem cell (hiPSC)-derived neurons, and murine pluripotent P19 cells—a neuronal precursor cell line used as an in vitro model system for neuronal differentiation. We developed an approach comprising automated particle detection, subtomogram averaging (STA), and spatial statistics combined with mass spectrometry (MS)-based proteomics to interrogate structures and potential functions of the lumenal particles in their native context. Our data provide important clues as to the particles' identities, show that MT lumenal particles are an integral part of the differentiated neuronal MT cytoskeleton, and that they may be involved in MT quality control.

## Results

### Organization of Lumenal Particles within MTs.

**Fig. 1.**
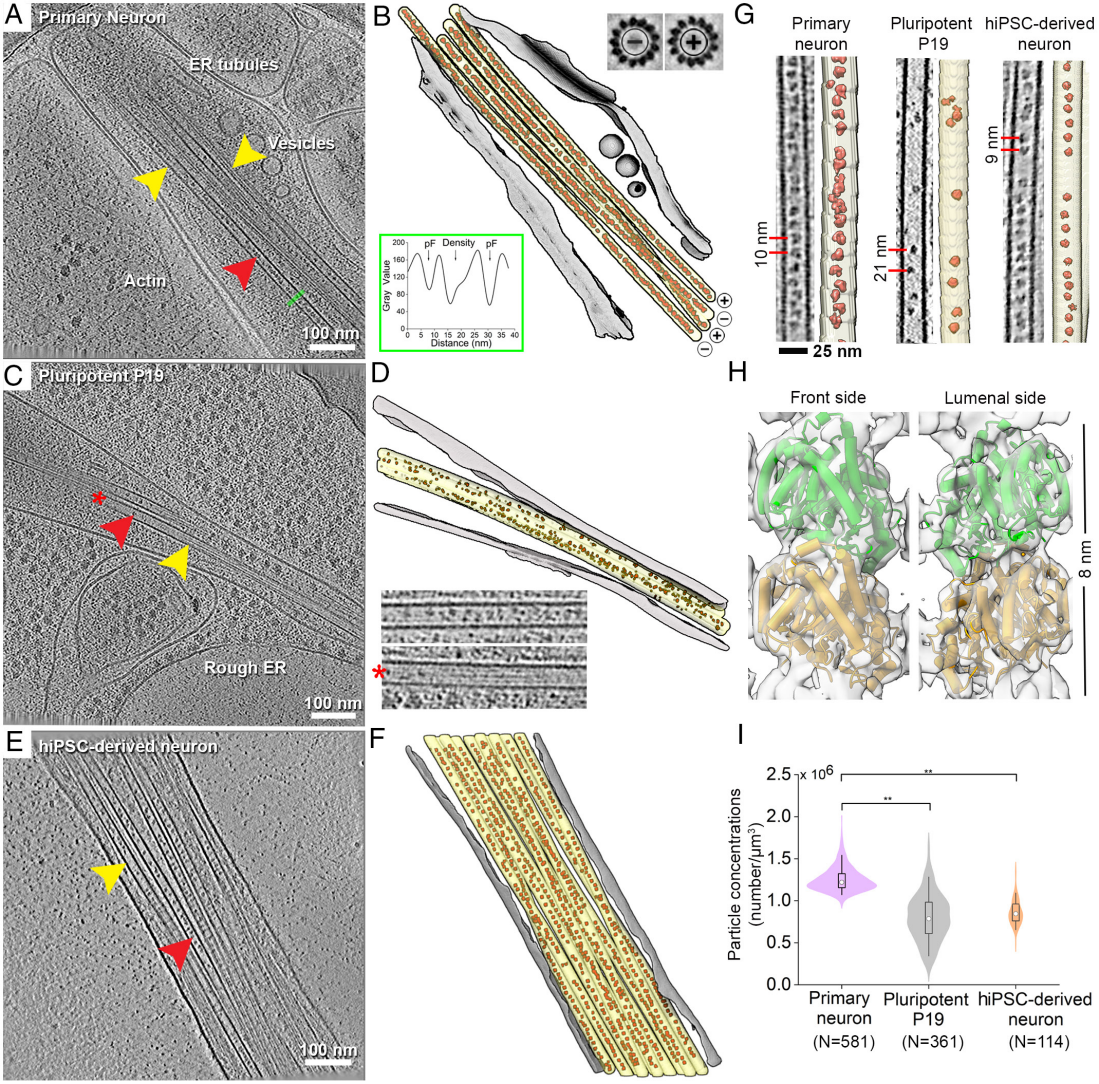
Organization of lumenal particles inside neuronal MTs. (*A*–*F*) Tomographic slices (*A*, *C*, E) and 3D annotations (*B*, *D*, *F*) of traced MTs (yellow arrowhead) with the detected lumenal particles (red arrowhead) and membranes (gray) for the indicated cell types: *A*, *B*. Primary neuronal process, slice thickness 9 nm; Insets in *B*: *Top Right*, subtomogram averages of individual MTs show different polarity. Below *Left*: line profile across a MT (green line in *A*) shows presence of extra density between pFs. (*C* and *D*) Pluripotent P19 process, slice thickness 6.8 nm. Inset in *D*: Zoom in of an empty MT marked by a red star compared to its neighboring MT. (*E* and *F*) hiPSC-derived neuronal process, slice thickness 4.25 nm. (*G*) Tomographic slices showing varying particle abundances in the indicated cell types. Segmentations of the MTs (yellow) and lumenal particles (red) are shown. Typical distances are indicated. (*H*) 8.2 Å in situ average of the 13-pF primary neuronal MTs, fitted with an atomic model of GDP-bound tubulin dimer [PDB:6dpv (42)]. α- and β-tubulin cannot be distinguished in the MT density map. The dimension of a tubulin-dimer indicated. (*I*) Quantification of the particle concentrations in lumenal volume represented as boxplot within a violin. Median values are marked by the white circle. Asterisks indicate Mann–Whitney test significance: **P < 0.01. N, number of MTs analyzed. See also *SI Appendix*, Figs. S1 and S2.

Cryo-ET of thin cellular processes (thickness <0.2 μm) of primary neurons, pluripotent P19 cells, and hiPSC-derived neurons revealed membrane-enclosed MT bundles (Fig. 1 *A*–*F* and Movies S1–S3). Globular particles were densely packed within the MT lumens of the primary and hiPSC-derived neurons (Fig. 1*G*). In comparison, particles were more sparsely distributed in the pluripotent P19 cells (Fig. 1*G*). Close inspection showed morphological similarity among lumenal particles across the cell types (Fig. 1*G*). Among those, the most notable feature was the presence of a ring-shaped particle, which was also recently described inside mouse dorsal root ganglion (DRG) and hippocampal neuronal MTs (21, 22).

We established the MT polarities in the imaged cellular processes of primary neurons using STA (*SI Appendix*, Fig. S1 *A*–*F*) (43). We obtained an 8.2 Å in situ MT map (Fig. 1*H* and *SI Appendix*, Fig. S1 *G*–*I*) with discernible secondary structural features showing MTs exclusively constituted of 13 protofilaments (pFs) and conforming to the consensus lattice architecture observed in mammals (44). We found both mixed (*Inset*: Fig. 1*D* and *SI Appendix*, Fig. S1 *A*–*C*) and uniform polarity orientations (*SI Appendix*, Fig. S1 *D*–*F*) in different processes, which indicate their identities as dendrites or axons, respectively (45). Lumenal particles were present within all MTs irrespective of their polarity (*SI Appendix*, Fig. S1 *B* and *E*).

To quantify the abundances of the lumenal particles, we developed an automated method for template-free detection of particles inside the segmented MT lumens (*SI Appendix*, Fig. S2 *A*–*C*) by employing discrete Morse theory segmentation and topological persistence simplification (46). In accordance with the visual inspection, the analysis revealed the highest concentration of lumenal particles in the primary neurons, as noted in earlier studies (17, 24), followed by that in hiPSC-derived neurons (Fig. 1*I*). In pluripotent P19 cells, the particle concentration distribution was broad (Fig. 1*I*), with neighboring MTs containing markedly different concentrations (*Inset*: Fig. 1*D*).

Next, we employed nearest-neighbor (NN) analysis (46) to describe the organization of the lumenal particles using the particle coordinates refined in RELION (47) (described below). We observed nonrandom distributions in all samples, with peaks at 8 to 10 nm representing the most common NN-distance (black dashed line, *SI Appendix*, Fig. S2 *D*–*F*) (17, 21, 25). This suggested the existence of statistically significant short-range order (≤10 nm), with the highest probability observed in primary neurons. Longer distances were not statistically significant, as they were also obtained from simulations of randomly distributed particles (gray shaded area, *SI Appendix*, Fig. S2 *D*–*F*). We statistically evaluated these spatial patterns for 2nd-order organization analysis at multiple distance scales using Ripley’s L function against complete randomness with numerical corrections for MT volumes (46) (*SI Appendix*, Fig. S2 *G*–*I*). Here, L values higher than random indicate more clustered organization than random at the given scale, and lower values indicate more uniform than random. We observed lower than random L values for lumenal particles in the primary and hiPSC-derived neurons, signifying their uniform distribution. This uniform organization is lost for scales larger than ~50 and ~40 nm, respectively. L values for P19 cells overlap with the complete randomness indicating that lumenal particles are randomly distributed. Our data and analyses thus agree with previously reported organizational differences of lumenal particles observed across different cell types (16, 17, 21, 22).

### 3D Morphologies of Lumenal Particles.

Next, we sought to elucidate the molecular morphologies of the lumenal particles for each cell type using STA (*SI Appendix*, Table S1). Marked structural and compositional heterogeneity, combined with the relatively small size of the particles (diameters in the range of 6 to 8 nm), posed a challenge for STA-based structure determination. In order to generate homogeneous particle groups for averaging, we subjected the particles obtained from template-free detection to several sequential rounds of 3D classifications until no new classes emerged (*SI Appendix*, Fig. S3). All class averages were obtained de novo, *i.e.* by reference-free analysis, validated against different references, and exhibited sufficient angular sampling (*SI Appendix*, Fig. S4 *A*–*G*). The approximate molecular masses of the lumenal particles were derived from the tomograms using the ribosomes as an internal reference, and found to be in the range of 200 to 400 kDa (*SI Appendix*, Fig. S5 *A*–*F*), in agreement with a previous analysis (22). The size and mass of these particles preclude classical motor–cargo complexes with molecular masses up to several mega Daltons (48), which have been previously hypothesized to be involved in the transport of proteins or mRNAs through the MT lumen (23, 24).

**Fig. 2.**
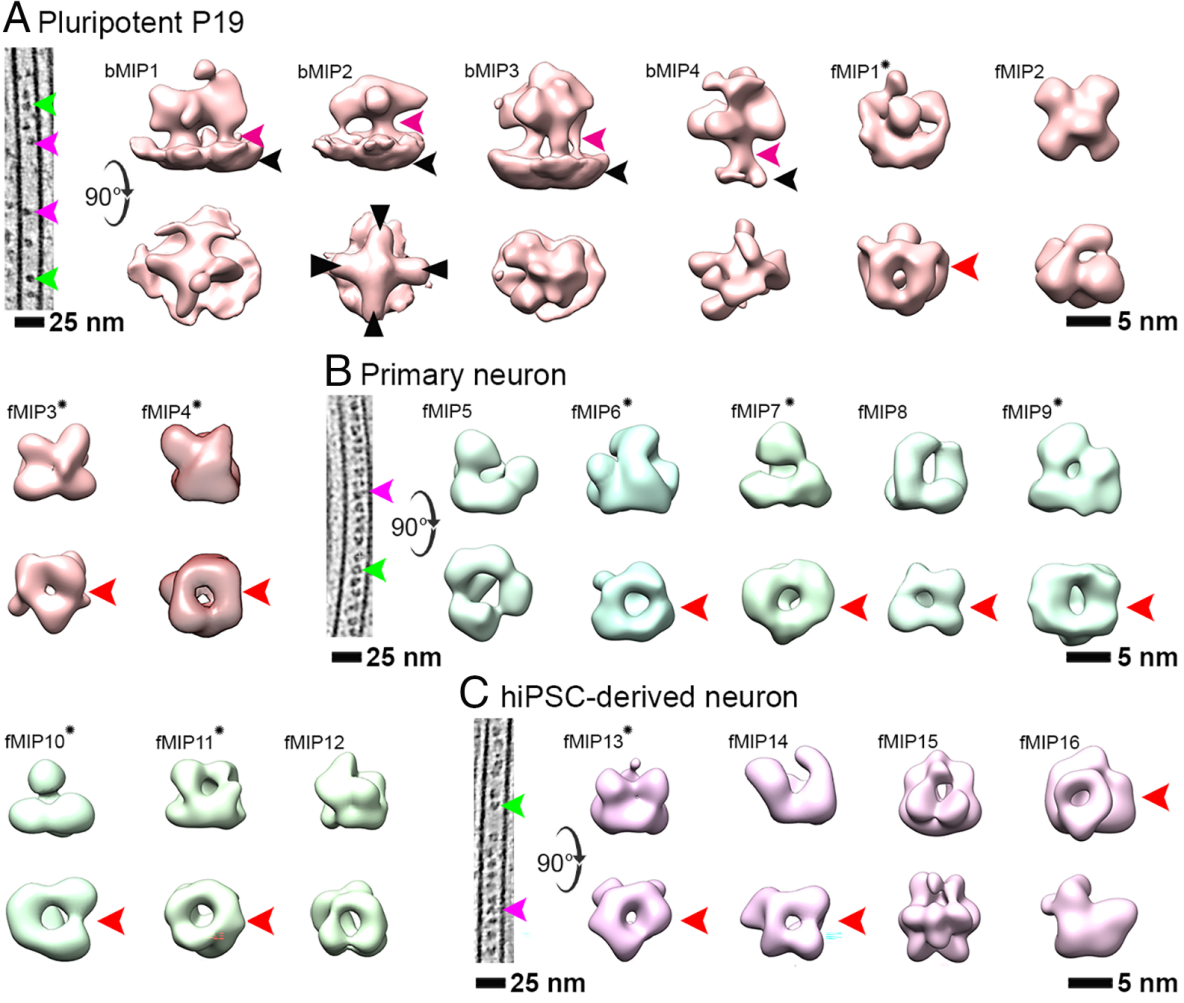
Subtomogram averages of MT lumenal particles. (*A*) *Left*, a tomographic slice of a pluripotent P19 MT showing bound (magenta arrowhead) and floating (green arrowhead) types of lumenal particles. Class averages of lumenal particles (*Right*; in brown) from pluripotent P19 cells. The curved arrow indicates 90° rotation of *Top* row views with respect to *Bottom* row. Bound particle classes (bMIP1-4) are indicated by their attachment to the MT wall (black arrowhead) with stalk like density (magenta arrowhead). Black triangles indicate four lobes of bMIP2. (*B* and *C*) *Left*, same as in (*A*) for the corresponding neuronal cell types. Class averages of lumenal particles found in primary neurons (*Right*; green) and hiPSC-derived neurons (*Right*; purple). Averages with ring-shaped scaffold present in all cell types are indicated by red arrowhead. Cage-like fMIPs with empty cores are marked with a star. Class averages are shown on the same scale. See also *SI Appendix*, Figs. S3 and S4.

Both visual and STA analyses of the pluripotent P19 tomograms indicated two types of particles based on their association with the MT wall: A subset of the particles showed connections with the MT wall, while others appeared floating inside the lumen (Fig. 2*A*). In the MT-wall bound category, the most notable class averages showed elongated particles with four lobes (Fig. 2*A*; black tringles). We termed these densities “bound MT inner protein 1” or bMIP1, and bMIP2, which we found to attach to the MT wall with stalk-like densities (pink arrowheads, Fig. 2*A*). The distance between the neighboring stalks was ~4 nm, equivalent to the size of a tubulin monomer. A third bound density (bMIP3) had a globular structure bound to the MT lumen wall via three stalk-like densities. A fourth-class average (bMIP4) bore similarity to bMIP2, but was bound to the MT lumen with one stalk and harbored an extra density on its top. All bMIPs were found exclusively in the pluripotent P19 cells, with bMIP2 and bMIP1 showing the highest and lowest abundances, respectively (*SI Appendix*, Fig. S6*A*).

The floating particles (fMIP) from all cell types appeared predominantly globular and were located centrally in the MT lumen (Fig. 2 *A*–*C*). STA showed that the densities were devoid of any putative stalks. Yet, the absence of a connecting density between the particles and the MT wall does not necessarily preclude a contact, because high flexibility can limit convergence into a discrete density in the average maps. This was suggested to be the case for the absence of a putative stalk density in the MIP average from mouse DRG neurons (red boxed, *SI Appendix*, Fig. S3) (21). Visual inspection indicated that most fMIPs contained a ring-shaped scaffold, a structural feature common to all the cell types investigated here (Fig. 1*G*) and previously reported for other mammalian neurons (21, 22). STA confirmed that the majority of fMIPs possess a ring-shaped scaffold (Fig. 2 *B* and *C*; red arrowheads), which we further substantiated by analyzing clustering of pair-wise cross-correlation (CC) values between the ring-shaped fMIPs (*SI Appendix*, Fig. S6*B*), implying high degree of similarity at the relatively modest resolution of the averages (20 to 32 Å, *SI Appendix*, Table S1). A few of the ring-shaped fMIPs exhibited a distinct cage-like topology with an empty core (Fig. 2 *A*–*C*; marked with star). Among them, fMIPs 4 (mouse P19), 11 (rat primary neurons), and 13 (human iPSCs) closely resemble the recently reported ring-shaped MIP density in mouse DRG neurons (CC values > 0.8) (21). The finding of a ring-shaped scaffold as one of the MIP components in various neuronal cell types underscores their potential general importance in MT biology. However, we found differences among the ring-shaped fMIPs that arise from the configuration and different sizes of the globular densities attached to the ring. Except for fMIP13, angular samplings of all the fMIPs were uniform suggesting these differences are less likely to originate from their orientation bias with respect to the tomographic missing wedge (*SI Appendix*, Fig. S4*A*). Non-ring fMIPs such as fMIP2 (in P19), 5 (primary neurons), and 15 (hiPSCs) are globular and topologically not related to each other. They could therefore be cell-type specific. fMIPs exhibited varied abundances. Among the ring-shaped fMIPs, fMIP1 of pluripotent P19 cells had similar abundance as fMIP 6 and 9 in primary neuron, while fMIP13 of hiPSC-derived neurons had the highest abundance of all (*SI Appendix*, Fig. S6*A*). In addition, unlike all other MIPs analyzed, fMIP13 particles are found to be organized with the most frequent NN-distance of 8 to 10 nm within the MT lumen (*SI Appendix*, Fig. S6*C*).

### Taxol Treatment Alters Lumenal Particle Distribution.

**Fig. 3.**
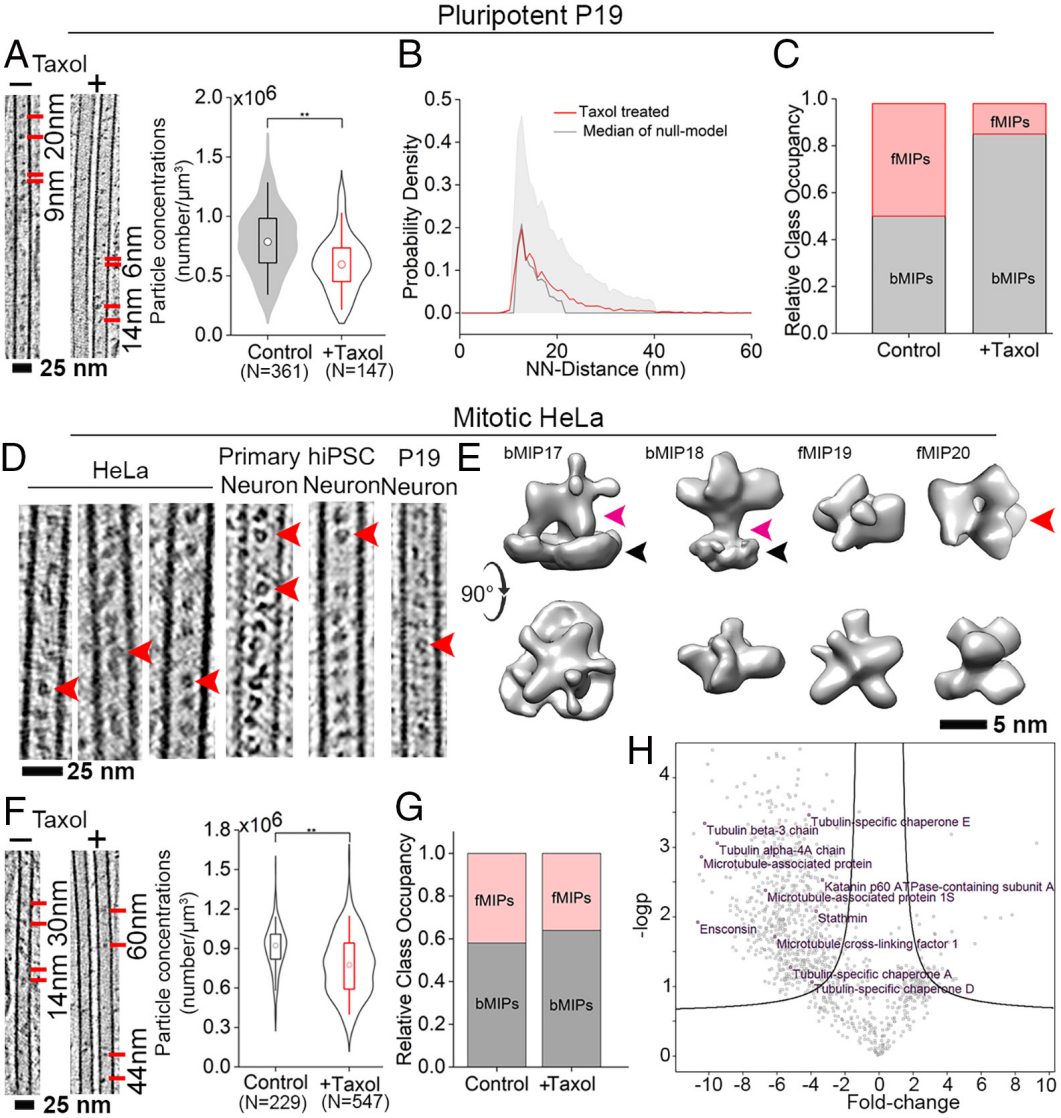
Taxol treatment reduces lumenal particle concentration and provides basis for AP-MS based particle identification. (*A*) *Left:* 6.8 nm thick tomographic slices of control (−) and Taxol-treated (+) pluripotent P19 cells showing lumenal particle distribution. Representative distances between particles are indicated. *Right*: Particle concentrations shown as combination of box-plot and data distribution within violin plot. The circle denotes median. Statistical significance using the Mann–Whitney test, **P < 0.01. N, number of MTs. Concentration for P19 from Fig. 1*I* is replotted here. (*B*) NN-distance distributions of the lumenal particles in Taxol-treated cells (red line). Null-model simulations representing complete spatial randomness are shown in black and shaded gray area indicates interval of confidence (IC) [5, 95] %. (*C*) Relative abundances of wall-bound (bMIPs) and floating (fMIPs) class averages in control and Taxol-treated pluripotent P19. (*D*) Tomographic slices of mitotic HeLa MTs (6.8 nm thick) in comparison to different neuronal cell types (9 nm thick) showing visually similar lumenal particle. Red arrowhead: ring-shaped floating particles. (*E*) Class averages of mitotic HeLa MT lumenal particles. Bound class of particle is indicated by their attachment to the MT wall (black arrowhead) with stalk like density (magenta arrowhead). The curved arrow indicates 90° rotation of *Top* row with respect to *Bottom* row views. Average containing ring-shaped scaffold is indicated by red arrowhead. (*F*) 9 nm thick tomographic slices of control and Taxol-treated mitotic HeLa cells showing lumenal particle distribution. Quantification of particle concentrations shown as in *A*. (*G*) Relative abundances of wall-bound (bMIPs) and floating (fMIPs) class averages in control and Taxol-treated mitotic HeLa. (*H*) Volcano plot showing fold-change of MAPs between control and Taxol-treated HeLa cells. MT-cytoskeleton related top hits (significant fold change *P* < 0.05) are highlighted. See also *SI Appendix*, Figs. S7 and S8.

In order to identify the molecular constituents of lumenal particles, we sought a way to modulate particle concentrations within the MT lumen to enable a proteomics-based analysis. We hypothesized that Taxol, whose binding is known to occur at the MT lumen (49) and to induce changes to the MT lattice spacing (50–52) could affect MIP binding or distribution. Therefore, pluripotent P19 cells were treated with 5 µM Taxol for 30 mins prior to vitrification and visualized by cryo-ET. Lumenal particle concentrations in the Taxol-treated samples were reduced by ~25% compared to the control (Fig. 3*A*; *SI Appendix*, Fig. S7 *A*–*D* and Movie S4). NN-distance measurement without considering particle identity and allowing for all possible neighbor combinations showed that lumenal particles became randomly distributed in Taxol-treated cells (Fig. 3*B*) compared to the short-range order observed in the control cells (*SI Appendix*, Fig. S2*E*). Classification revealed a global reduction in the fMIPs and an increase in bMIPs following Taxol treatment. In fact, from a 1:1 ratio of fMIPs to bMIPs observed in the control cells, bMIP1 became almost exclusive in Taxol-treated P19 cells (Fig. 3*C* and *SI Appendix*, Fig. S7*E*). Congruently, the probability of a bMIP1 particle having another bMIP1 as its neighbor at 8 nm distance increased compared to the control cells (*SI Appendix*, Fig. S7*F*). Therefore, Taxol treatment offered an opportunity to probe the identity of MIPs.

### A Subset of fMIPs Identified as Putative Tubulin Binding Cofactors.

We leveraged our observation of Taxol-induced reduction in lumenal particle abundance as an experimental condition to identify Taxol-sensitive MAPs using a differential enrichment of tubulin-interacting proteins in control and Taxol-treated cells by affinity purification-MS (AP-MS) based proteomics. Toward that end, we opted to use nonneuronal HeLa Kyoto cells that offered a technical advantage as they can be grown in sufficient quantities suitable for pulldowns. Mitotic HeLa (in contrast to interphase cells) expressing C-terminally GFP*-*tagged β-tubulin (26, 53) exhibited lumenal particles with similar abundances as in pluripotent P19 cells (*SI Appendix*, Fig. S8*A*). Importantly, no statistically significant difference was observed in lumenal particle concentrations between tagged and untagged mitotic HeLa cells (*SI Appendix*, Fig. S8*B*). Visual inspection showed that the HeLa lumenal particles share morphological similarities, including the ring-shaped particles, with those of the neuronal cells examined in this study (Fig. 3*D*). STA of the mitotic HeLa lumenal particles indeed provided class averages (bMIP17-18) resembling bMIP1, 4 and (fMIP19-20) resembling fMIP2, 3 observed in P19 cells (Fig. 3*E* and *SI Appendix*, Fig. S8*C*).

Taxol treatment globally reduced the abundance of lumenal particles in the mitotic HeLa (Fig. 3*F* and *SI Appendix*, Fig. S8 *D*-*G*), similar to the observed effect in P19, but led to loss of both bMIPs and fMIPs almost at equal proportions (Fig. 3*G*). Therefore, β-tubulin was isolated by affinity purification using an anti-GFP antibody from intracellularly crosslinked control (DMSO-treated) and Taxol-treated mitotic HeLa cells (*SI Appendix*, Fig. S8*H*). Cross-linking ensured preservation of information on transient MT-interacting particles during the isolation procedure. Differentially enriched proteins were identified by MS. We obtained a total 168 MT cytoskeleton-related proteins (see MS data table, *SI Appendix*, Fig. S8*I*), including 29 MAPs known to bind the MT outer surface, such as Ensconsin, MAP7D1, MAP4, and MAP1S (Fig. 3*H* and *SI Appendix*, Table S2), whose levels reduced significantly (*P* < 0.05) in the presence of Taxol. Loss of MAPs could be due to an expected expansion of the MT lattice induced by Taxol binding, as suggested by recent in vitro studies (50–52, 54). Notably, the GFP-tag on β-tubulin pulled down several β-tubulin binders, such as tubulin-binding cofactor (TBC) D and TBCA whose levels were modestly reduced in the presence of Taxol. Since TBCD and TBCA are known to be cytosolic, both cytosolic and MT-associated fractions are equally susceptible to pull-down using the tag on β-tubulin, also upon Taxol treatment. In contrast, a significant reduction was observed for the α-tubulin binder TBCE, which indicated close association of the α-tubulin with β-tubulin as would be expected within the MT lattice/lumen and that is displaced upon Taxol treatment (within 1 nm distance dictated by the DSS spacer length).

**Fig. 4.**
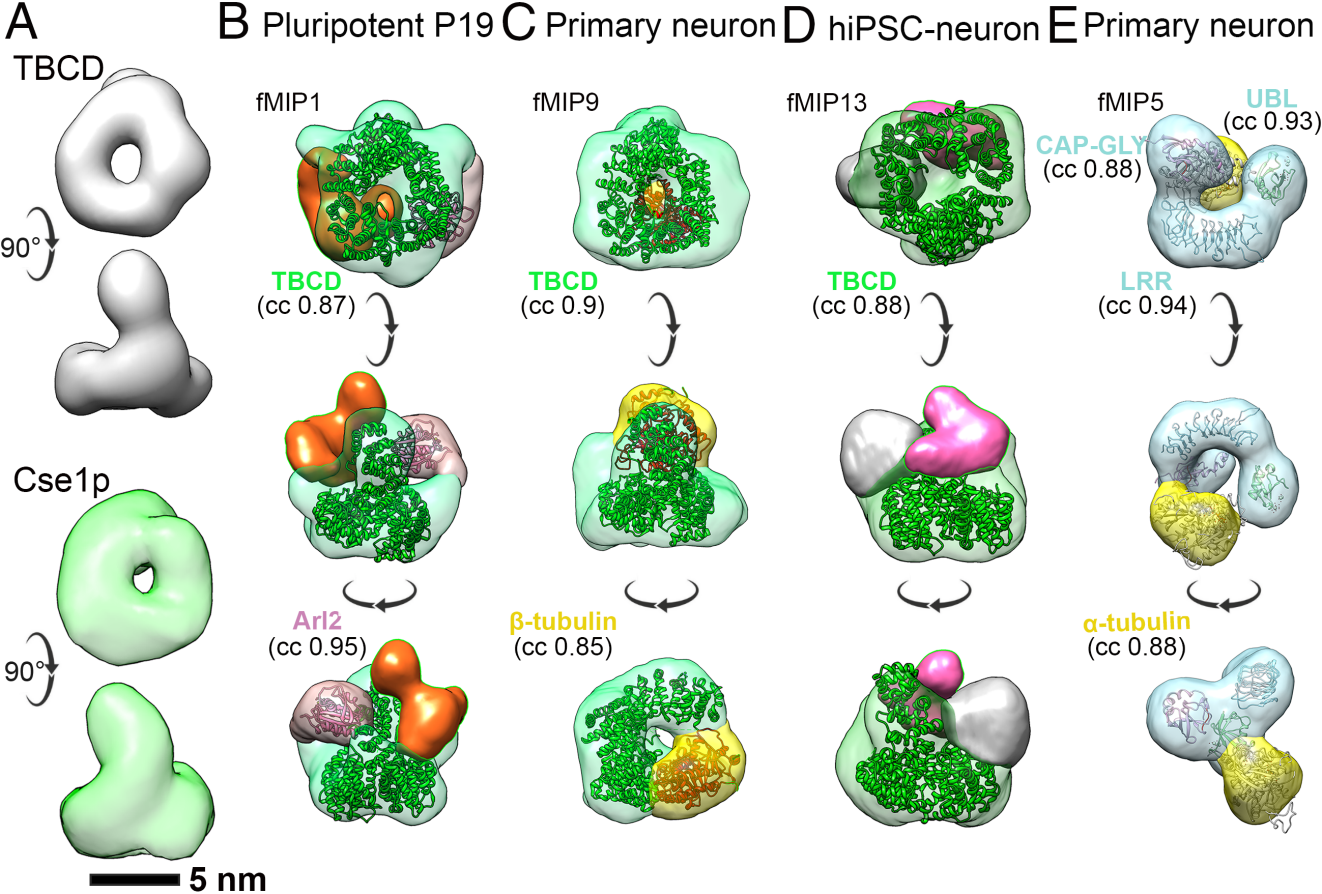
A subset of fMIPs resemble tubulin-binding cofactors (TBCs). (*A*) *Top Right*: single particle EM map of TBCD (EMDB-6390) (57) showing ring-shaped base. *Bottom Right*: low pass filtered map of Cse1p (PDB: 1Z3H) (59) at 25 Å. (*B*) Segmented density map of fMIP1 from P19 cells shown in three views. The ring-shaped base (green) is fitted with HEAT repeats of Cse1p. Light pink density is fitted with the GTPase, Arl2 [PDB: 1KSH (62)]. Remaining density (orange) is unassigned. (*C*) Segmented map of fMIP9 from primary neurons. The ring-shaped base (green) is fitted with Cse1p HEAT repeats. Yellow density is fitted with β-tubulin [PDB: 1TUB (66)] based on biochemical evidence. (*D*) Segmented map of fMIP13 from hiPSC-derived neurons. The ring-shaped base (green) is fitted with Cse1p HEAT repeats. Remaining densities (gray and pink) are unassigned. (*E*) Segmented map of fMIP5 from primary neurons. The U-shaped base (blue) is fitted with LRR [PDB: 3RJ0 (65)] and two globular head domains are fitted with UBL [PDB: 4ICV (67)] and CAP-Gly [PDB: 1WHG (64)] domains of TBCE. Yellow density is fitted with α-tubulin. CC values from rigid body fitting of each model low pass filtered to 25 Å are indicated. See also *SI Appendix*, Figs. S6 and S9.

We next systematically analyzed all 168 hits belonging to the Gene Ontology (GO) term of cytoskeleton-related proteins by examining their structures (if available) or structural predictions (55). Our analysis showed that most bona fide MAPs found in this study along with the MAP6, a suggested component of neuronal MT lumen (41), are predicted to be disordered and therefore unlikely to correspond to our globular density maps (*SI Appendix*, Fig. S9 and Table S2) (21). Notably, we did not find TAT, the most commonly perceived component of lumenal particles, in our AP-MS hits even though mitotic MTs are known to be highly acetylated, similar to neuronal MTs (56). We next low-pass filtered the models and available structures of the candidates to the resolution of the density maps generated for the lumenal particles for comparison (*SI Appendix*, Table S2). Interestingly, ring-shaped scaffolds of the cage-like fMIPs (Fig. 2 *A*–*C*, marked with star) were found to bear high similarity (CC of >0.8) to the 24 Å negative stain EM-derived map of TBCD (mass 132 kDa, EMD-6390/6392) (57), involved in folding and degradation of β-tubulin (Fig. 4*A*) (58). We used the crystal structure of the yeast Cse1p (PDB:1Z3H) (59) that structurally mimics TBCD (57), filtered to 25 Å resolution to fit into our ring-shaped EM density using a rigid body fitting protocol (Fig. 4*A* and *SI Appendix*). The distinct alpha-solenoid ring structure of the Cse1p made up with HEAT repeats fitted the ring-shaped base of the fMIP1, 9, and 13 (CC > 0.85, green, Fig. 4 *B*–*D*). Biochemical studies show that TBCD can exist in a number of distinct complexes: TBCD:ADP ribosylation factor-like protein 2 (Arl2) (60), TBCD:Arl2:β-tubulin (61), TBCD:β-tubulin (61), TBCD:α-β tubulin (61), TBCD:TBCE:Arl2 (57), and TBCD:TBCE:Arl2:β-tubulin (57). In case of fMIP1, a model of Arl2 [PDB: 1KSH (62)] fitted well (CC 0.95) within the globular density (light pink), suggesting a complex between TBCD and Arl2 (Fig. 4*B*) with a minimum projected mass of ~150 kDa. A portion of the density (orange) remained unassigned. A β-tubulin density fitted in the remaining globular density of fMIP9 with a CC of 0.85 (yellow, Fig. 4*C*). Therefore, fMIP9 tentatively represents a binary complex between TBCD and β-tubulin with a projected mass of ~180 kDa. In case of fMIP13, portions of the density (gray and magenta) remain unassigned (Fig. 4*D*). The mass of these 3 putative complexes correlates with our mass measurements from the tomograms (*SI Appendix*, Fig. S5*F*). The U-shaped density with two globular heads of fMIP5 (cyan, Fig. 4*E*) bore structural resemblance to the negative stain EM-derived map of TBCE that binds α-tubulin (EMD-2447) (63). As in the TBCE density map, the globular heads of fMIP5 fitted the Cap-Gly [PDB: 1WHG (64)] and UBL domains (PDB: 4ICV), while the curved segment between the globular heads likely corresponds to the TBCE LRR domain (63). The curved segment fitted TLR4 (57) that structurally mimics the TBCE LRR domain [CC 0.94, PDB: 3RJ0 (65)]. The Cap-Gly domain binds to a globular density in our map that can accommodate α-tubulin [yellow, with CC 0.88, Fig. 4*E*, PDB: 1TUB (66)], and importantly places the tubulin binding loop close to the tubulin C-terminus. Based on the above assignments, we suggest putative identities for 9 ring-shaped lumenal particles (fMIP1, fMIP3-4, fMIP6-7, fMIP9-11, and fMIP13) and one non-ring-shaped complex (fMIP5), out of the 16 fMIPs we characterized in the neuronal cells, to be TBCs bound to tubulin. These accounted for a high fraction of MT lumenal particles in the different neuronal cell types (*SI Appendix*, Fig. S6*A* and Table S1, up to 75% of all fMIPs in primary neuron).

### MT Lumenal Particle Abundance Increases in Neuronal Differentiation.

**Fig. 5.**
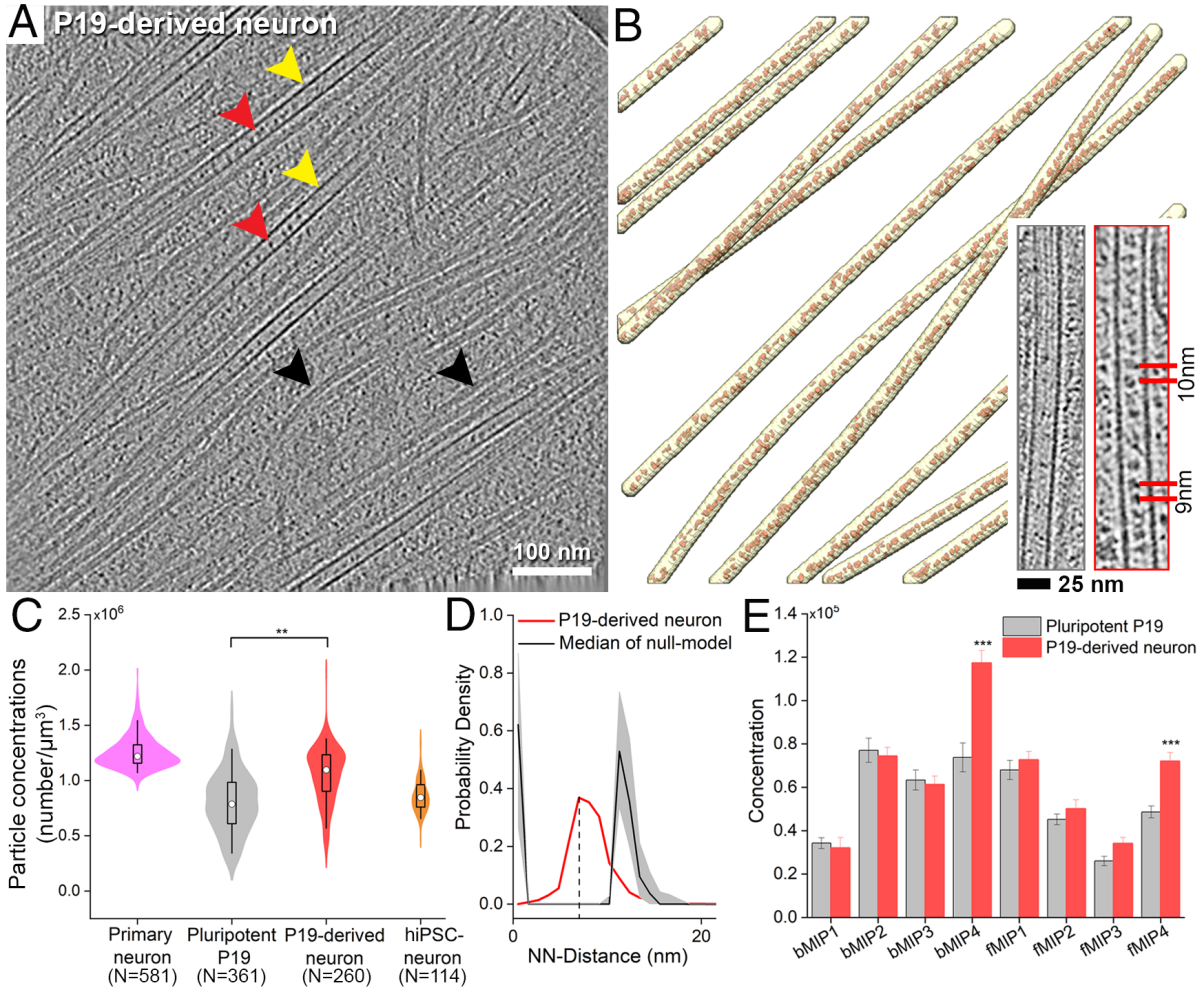
Induction of neuronal differentiation increases abundance of MT lumenal particles. (*A*) 9 nm thick tomographic slice of a P19-derived neuronal process at 10 d in vitro differentiation showing MTs (yellow arrowhead) containing lumenal particles (red arrowheads), and parallel array of Intermediate filaments (black arrowheads). (*B*) 3D rendering of traced MTs (yellow) and lumenal particles (red). *Inset*: tomographic slices of a pluripotent P19 MT (*Left*) and a P19-derived neuronal MT (*Right*) showing change in particle abundance and distribution. (*C*) Quantification of the particle concentrations represented as boxplot within a violin. For comparison, concentrations for primary, P19 and hiPSC-derived neuron from Fig. 1*I* are replotted here. Median values are marked by a circle. Asterisks indicate Mann–Whitney test significance: **P < 0.01. N, number of MTs. (*D*) NN-distance distributions of the lumenal particles in P19-derived neurons. The black dashed line indicates most represented NN-distance, the gray shaded area indicates IC [5, 95] %. (*E*) Comparison between the concentrations of each class average before (gray) and after differentiation (red). Error bar indicates SEM. Statistical significance ***P < 0.001, obtained by the two-sample *t* test.

Observations of periodically and densely decorated MT lumens particularly in rodent and hiPSC-derived neurons (Fig. 1*G*), as well as in other mammalian and insect neurons (17, 21–24), raise a question as to whether such decoration represents a characteristic of differentiated neuronal MTs. To address this question, we took advantage of the ability of pluripotent P19 cells to differentiate and develop neuronal processes in vitro (68). Neurospheres generated after aggregation of the P19 cells upon exposure to 0.5 µM retinoic acid (RA) were allowed to differentiate for about 1 wk. Neuron-like cells were selectively grown using a DNA synthesis inhibitor (*SI Appendix*). When these in vitro differentiated neurons were imaged using cryo-ET, their thin processes revealed parallel arrays of MTs (Fig. 5 *A* and *B*). Unlike pluripotent P19 cells, the MT were frequently observed embedded in a dense intermediate filaments network (Fig. 5*A*) (68, 69). The cryo-ET data revealed a significantly higher concentration of tightly packed lumenal particles relative to their parental state, arranged in a periodic array with 8 to 10 nm NN-distance (Fig. 5 *B*–*D*; *Inset*). Thus, emergence of dense and ordered arrays of lumenal particles occurs after in vitro neuronal differentiation and could be a characteristic of neuronal MTs (17, 20–24). STA further showed enrichment of P19-specific particles in the MT lumen after differentiation, with a significant increase in the concentrations of bMIP4 and fMIP4 (Fig. 5*E*). In particular, lumenal particles with an ability to bind to the MT wall (such as bMIP4) and/or invoke tubulin proteostasis (fMIP4) could reflect emergent properties of the MT cytoskeleton in adaptation to neuron-specific functions.

### Lumenal Particle Abundance Correlates with MT Curvature, Lattice Defects, and Freshly Polymerized Plus Ends.

**Fig. 6.**
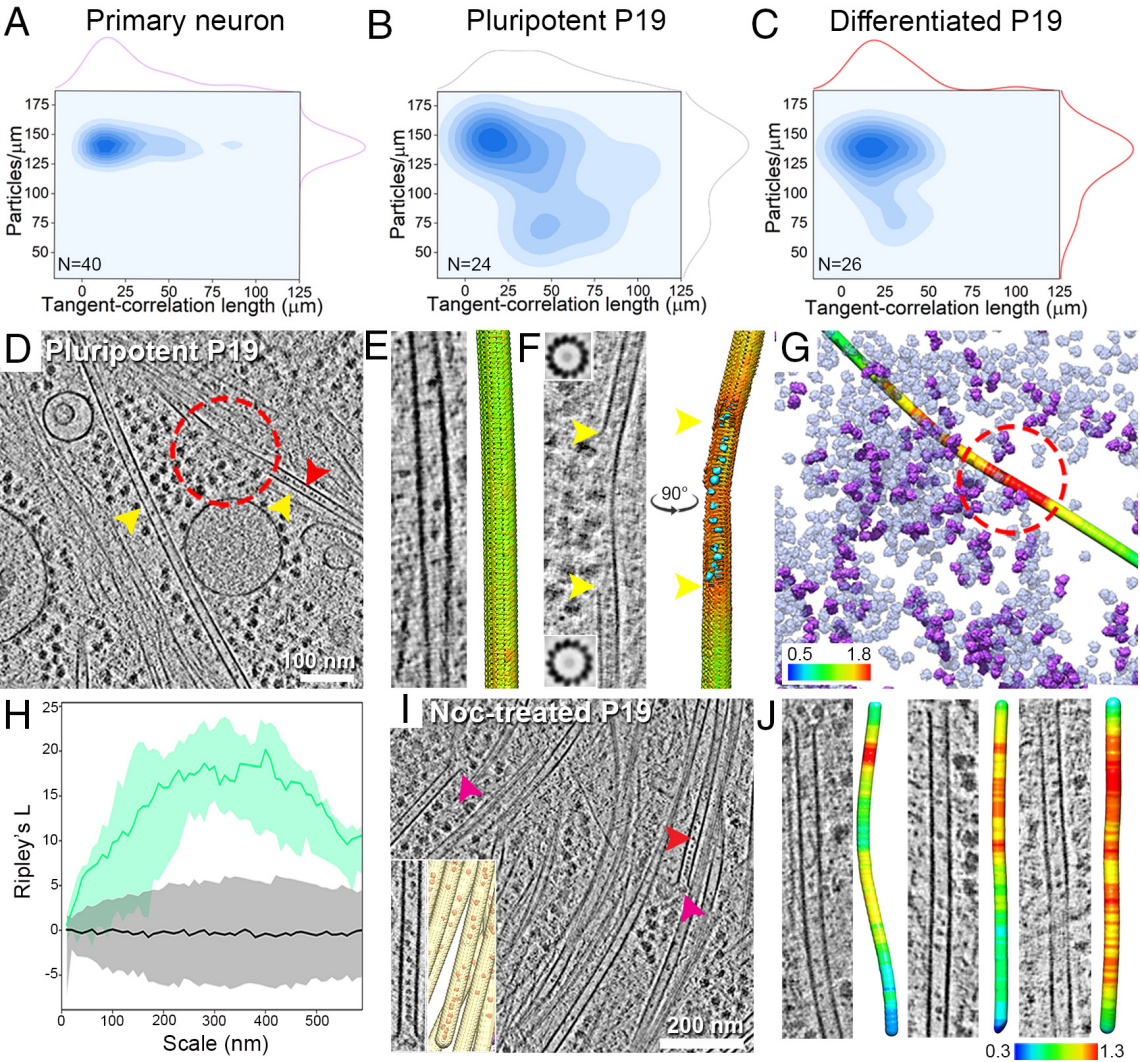
Lumenal particles abundance correlates with MT curvature, lattice breaks, and plus ends. (*A*–*C*) Distribution plots of tangent-correlation lengths (^a^L_p_) and mean particle abundance for the indicated cell types shown on the margins. Correlation between ^a^L_p_ and particle abundance indicated as contours. N, denotes numbers of tomograms analyzed. (*D*) 6.8 nm thick tomographic slice of a pluripotent P19 cell showing a broken MT (red circle). Yellow arrowheads indicate MTs. The red arrowhead indicates lumenal particles. (*E*) A tomographic slice of an intact pluripotent P19 MT and corresponding lattice map of tubulin monomers. Subtomograms are represented as arrows and color coded by CC values. Green represents highest CC. (*F*) Similar to *B*, but for a partially broken MT lattice (between yellow arrowheads). The lattice map shows missing subtomograms in the damaged regions. 3D-surface rendered lumenal particles (cyan) mapped back in the exposed MT region. STA confirmed 13 pFs in the flanking regions of severed lattice (2D cross sections of averages at *Top* and *Bottom*). (*G*) Surface rendering of the broken MT in D color-coded according to lumenal particle occupancy. Red represents highest abundance. Ribosomes shown as transparent violet. Ribosomes that form polyribosome shown in violet. (*H*) Bivariate Ripley’s L function for the particles clusters found at the partially broken MT lattices (N = 15 MT breakpoints; green line). The black line indicates median of the L function for a set of random particle distributions and the gray shaded area represents the IC [5, 95] %. (*I*) 6.8 nm thick tomographic slice showing particle distribution inside freshly polymerized MTs after Nocodazole (Noc) treatment and wash-out. Arrowheads indicate open-end of MTs (magenta) and lumenal particles (red). Inset is an enlarged image and 3D rendering of a MT open-end. (*J*) Representative slices of open-end (*Top* side) of freshly polymerized MTs in pluripotent P19 cells with corresponding surface rendering color-coded with spatial occupancy map of lumenal particles along the MT length (red represents highest abundance). See also *SI Appendix*, Figs. S5, S10, and S11.

We next asked whether the enrichment of lumenal particles in differentiated cells is linked to any measurable MT properties. Cryo-tomograms revealed curved MTs in neuronal processes, with segments exhibiting sinusoidal trajectories (*SI Appendix*, Fig. S10*A*). We first quantified the extent of MT curvature using the tangent-correlation length (^a^L_p_, *SI Appendix*, Fig. S10*B*) (26). ^a^L_p_ in both primary and hiPSC-derived neurons indicated high curvature with mean values of 28.4 ± 3.4 µm and 21.7 ± 4.2 µm, respectively. In contrast, pluripotent P19 MTs were less curved with a broad ^a^L_p_ distribution and a mean of 41.6 ± 5.33 µm (Fig. 6 *A*–*C*: *Top* margins, *SI Appendix*, Fig. S10*B*). Upon P19 differentiation in vitro, the distribution narrowed and centered on 25.9 ± 3.9 µm, at par with the curvatures found in primary neurons. Examining the correlation between MT curvatures and lumenal particle concentrations showed that in primary neurons, highly curved MTs contained higher particle concentrations (Fig. 6*A*). A similar trend was observed in hiPSC-derived neurons, for which only 4 data points could be measured. In pluripotent P19 cells, a distinct segregation into two populations was observed where highly curved MTs contained high particle concentrations (similar to primary neurons) (Fig. 6*B*) and vice versa. Upon differentiation, the P19 cells developed a trend similar to the primary neurons (Fig. 6*C*). This correlation suggested that lumenal particles, predominantly bMIPs including bMIP4 (Fig. 5*E*), preferentially localized within curved MTs, potentially contributing to stabilization of curved MT lattice in a manner similar to that suggested for lumenal MAP6 (41).

High MT curvature could lead to breaks (26), which may represent entry points into the lumen, as was suggested for TATs (70). We therefore next examined the abundance of lumenal particles with respect to partially broken and exposed MT lattice regions in pluripotent P19 cells. We pinpointed several partially broken MTs showing loss of one or several pF segments (Fig. 6*D*). A “lattice map” of one such representative case obtained by STA, where individual subtomograms were mapped back to the original positions in the tomogram and color coded according to the CC values (Fig. 6 *E*–*F*), showed that CC-values flanking the broken site were lower, in accordance with a disordered lattice. The pF number in the regions flanking the broken lattice was 13, indicating that pF homogeneity is maintained near large defects *in vivo,* in contrast to the known pF heterogeneity observed in vitro (Fig. 6*F*). Heterogeneity in pF number has been suggested to weaken the MT lattice and as a result may induce the formation of defects and/or kinks (71). Lumenal particles in these regions were counted and represented as a color-coded spatial occupancy map, highlighting the presence of a “microcluster” flanking the broken region compared to the intact part (Fig. 6*G*, red segment). We used bivariate Ripley’s L function to validate statistical significance of such clustering (46). Distances between the particles and break points were measured in 15 such instances and compared with complete randomness using Monte Carlo simulation. The experimental median of the L function displayed a bell-shaped curve with positive values (green line, Fig. 6*H*) that is distinct from the random simulation (gray area, Fig. 6*H*), indicating clusters of a size of ~300 nm around the lattice breaks were statistically significant.

We next tested whether microclustering also occurs at the open MT ends representing another possible entry point for lumenal particles. To this end, MT polymerization was initiated in pluripotent P19 cells by a brief Nocodazole treatment followed by drug washout (*SI Appendix*) (26). While particle concentration was overall lower in newly polymerized MTs compared to the control (*SI Appendix*, Fig. S11*A*), lumenal particles clustered near the MT open-ends (*Inset*: Fig. 6*I*) in variable sizes as revealed by the red segments in the spatial occupancy maps (Fig. 6*J* and Movie S5). We statistically evaluated such clustering using the previously described bivariate Ripley’s L function, which revealed a weak tendency of the particles to cluster with domain size up to 200 nm from the MT open-end (*SI Appendix*, Fig. S11 *B* and *C*). The particles were more tightly packed with a mean interparticle distance of 9.6 ± 3.4 nm within ~100 nm from the MT open-end compared to distal regions (mean distance 14.3 ± 6.7 nm) (*SI Appendix*, Fig. S11*D*). STA showed that freshly polymerized MTs accumulated similar classes of both bMIPs and fMIPs as the control P19; among the class averages, concentrations of bMIP3, fMIP1, 2, and 4 were significantly reduced (*SI Appendix*, Fig. S11*E*). Visual inspection showed that both bMIPs and fMIPs are part of the clusters observed near the MT open end. Our data thus suggest that particles can access the lumenal space and form small clusters at MT lattice breaks or via the open-end of the polymerizing MTs. The presence of such clusters might impact nascent MT-tip during polymerization or sites of lattice damage through tubulin lattice stabilization.

## Discussion

The presence of lumenal particles inside MTs has been known for decades (13–17, 72). Yet, our understanding of the molecular components contributing to the formation of these particles and their functions remained elusive. It is an open question whether MT lumenal particles represent novel types of MAPs involved in modulating MT stability, or whether they could be proteins or mRNA that are stored or transported inside MTs (23, 24). We leveraged in situ cryo-ET combined with STA and targeted proteomics to extract structural, spatial, and functional information about MT lumenal particles of various neuronal cells.

The periodic decoration of the MT lumen by particles appears to be a key feature of neuronal MTs (Fig. 5*E* and *SI Appendix*, Fig. S2 *D*–*F*) (17, 21–24). Such decorations could endow properties such as stability to neuronal MTs (73). Extensive mechanical forces are expected to be exerted on MTs during neuronal development (74), trafficking (2), and axon migration (75). As a force-bearing element, neuronal MTs are subject to bending and coiling, resulting in highly curved appearance in situ (26, 41) (*SI Appendix*, Fig. S10 *A* and *B*). The densities we resolve for bMIPs (Fig. 2*A*) are well placed to structurally stabilize curved MT lattices by crosslinking several pFs together via multiple stalk-like densities (positioned 4 nm apart), in analogy to MIPs related to MT doublets in motile cilia and flagella that function to stabilize the MT lattice (29–33). For cytoplasmic MTs, MAP6, an intrinsically disordered protein with three MT binding Mn modules is known to stabilize curved neuronal MT lattice in a similar manner from the lumenal side (41). The emergence of curved MTs during the course of neuronal differentiation of P19 cells with concomitant enrichment of bMIPs further supports this hypothesis (Figs. 5*E* and 6 *A*–*C* and *SI Appendix*, S10B). Furthermore, in the pluripotent P19 cells, a higher concentration of lumenal particles correlated with more curved MTs and vice versa (Fig. 6*B*). Therefore, bMIP localization or enrichment seems to occur selectively in MTs that experience larger mechanical forces and suggests their protective role in maintaining MT lattice architecture. Taken together, bMIPs may add another layer to the existing mechanisms that regulate material properties of neuronal MT along with structural MAPs and a complex tubulin-code (76).

Our proteomics data combined with structural modeling suggest that fMIPs could be TBCs bound to monomeric tubulin (Fig. 4 *B*–*E*). However, considering the limited resolution of the fMIP maps and the fact that our proteomics analysis was performed on HeLa cells to obtain sufficient material for the measurements, a conclusive demonstration would require further work. Nevertheless, TBCs are ubiquitously found in all cells and are essential for “tubulin proteostasis,” including folding of tubulin monomers, formation of tubulin heterodimers and for tubulin degradation (77). Their presence within the lumen could have interesting implications, especially since tubulin biogenesis and the localization of its folding machinery in neuronal cells, particularly axons, is not well understood (78). Tubulins must be present in sufficient amounts in distal axons to support nucleation and dynamics of MTs for the maintenance of synapses and growth. However, axonal processes contain low numbers of ribosomes, in agreement with low protein synthesis rates along axons (79). Furthermore, tubulins are transported from the cell body to distant neurites/axons at a slow speed of 0.1-3 mm/day (78, 80). Therefore, it is tempting to consider a mechanism wherein TBC-bound tubulins (e.g. fMIP9, 5) packed within the MT lumen could provide fresh dimers for MT lattice repair and growth in distal regions of neurons. They could coordinate with locally translating ribosomes to replenish the tubulin pool to support new MT nucleation crucial for growth-cone expansion or synaptic stability. In addition, TBCD:Arl2 complex (e.g. fMIP1) might ensure maintenance of the MT cytoskeleton integrity (81) by removing damaged tubulins from the lattice (60). Both TBCE and TBCD could recycle or degrade misfolded tubulins that pop out of the lattice due to MT bending or severing, along with TBCB and TBCA, respectively. Removal of the extruded β-tubulins by TBCD is deemed crucial since they form cytotoxic aggregates (82). Notably, we have not detected TBCB (27 kDa) or TBCA (13 kDa) in our class averages possibly due to their small sizes. Taken together, fMIPs might impact MT homeostasis locally and support MT-dependent processes such as growth cone expansion, axon branching, and maintenance of synapses (83).

Long-lived neuronal MTs are suggested to accumulate nanoscale lattice defects due to extensive synaptic trafficking, force-bearing during neuronal growth and regulated action of the MT severases (25, 26, 84). Generally, depolymerization of defective MTs, either due to their intrinsic property or by the action of severing enzymes, and polymerization of a new set of MTs has been regarded as the main mechanism for maintaining a functional MT network. However, such wholesale replacement mechanism in long neuronal processes could disrupt neuronal function. In such scenario, bMIPs could stabilize the partially severed MT lattice by forming cluster around the defects and holding the remaining pFs together (Fig. 6*G*), thereby providing sufficient time for MT self-repair through fresh tubulin incorporation. This proposed mechanism is in agreement with in vitro studies showing that spastin and katanin generate partially severed MTs by extracting damaged tubulins from the MT lattice, that in turn acquire fresh GTP–tubulin from solution, rejuvenate MTs, and amplify the MT arrays (85). On the other hand, fMIPs could provide fresh tubulins for their incorporation in the damaged lattice. In this case, TBCD, TBCE, Arl2, and tubulins, come together to form a catalytic complex that produces tubulin-dimers (57). However, dimer formation is expected to be very slow inside the lumen, due to the space constraint and slow one-dimensional diffusion inside the lumen (86). Therefore, catalytic complex would form efficiently only when particles are released through the lattice defects. This process presents an optimal way to self-repair, while maintaining the complex “tubulin code” required for long-lived neuronal MTs (83). Our findings could further shed light on the potential role of impaired tubulin proteostasis resulting in defective MT networks observed in severe neuro-developmental disorders such as giant axonal neuropathy (87), hyperparathyroidism (88), inherited early-onset encephalopathy (89), and infantile neurodegeneration (90).

In summary, we elucidate the native organization and morphology of neuronal MT lumenal particles and suggest that a subset represents a number of TBC complexes. While the conclusive demonstration of the existence and role of TBCs in neuronal MT lumens will require further targeted studies, we hypothesize that the MT lumen could serve as a transport channel for delivering fresh tubulins (23, 24) that potentially play a role in maintaining MT lattice integrity under compressive or transport-related forces in neurons by invoking local tubulin homeostasis, or that provide the essential building blocks for MT growth at distal end of neuronal processes.

## Materials and Methods

### Cell Culture and Grid Preparation.

Rat hippocampal neurons derived from embryonic rats (E17-21), murine pluripotent P19 cells, hiPSC-derived neurons, and HeLa cells were cultured in appropriate cell culture media in dishes containing pretreated EM grids according to methods described in *SI Appendix*. Samples were vitrified by plunge-freezing. Tilt series for all the cells were collected on a Titan Krios (Thermo Fisher Scientific) operated at 300 kV.

### MT Tracing and Curvature Measurement.

MT segmentations were performed in Amira software v.6.2.0 (Thermo Fisher Scientific) as described before (26). The coordinates of the traced filaments were resampled in MATLAB (MathWorks) to obtain equidistant points. MT curvature was determined by measuring the tangent-correlation length as described before (26).

### Density Tracing and Particle Picking.

Tomograms were Gaussian low-pass filtered at σv = 0.5 pixels and the MT lumen segmented based on the MT centerline tracing. PySeg (46) was used to pick particles in a template-free manner as detailed in *SI Appendix*.

### Subtomogram Averaging.

Subtomogram analysis for lumenal particles was performed using RELION (version 3.0.5) following published protocols (91). During the refinement, particle half-sets were processed independently. Unless stated explicitly, all refinements were performed de novo, i.e. without the use of external references. Datasets corresponding to different cell types were processed separately. Extensive classifications were performed to derive maps of different particle populations.

## Supplementary Material

Appendix 01 (PDF)

Dataset S01 (XLSX)

Movie S1. Tomographic slices of a primary hippocampal neuronal process showing the ultrastructure of the MT cytoskeleton. Related to Figure 1A, S1A. Color coding, MT: yellow, membrane: grey and lumenal particles: red.

Movie S2. Tomographic slices of a pluripotent P19 cellular process showing the ultrastructure of the cytoplasm containing MTs. Related to Figure 1C. Color coding, MT: yellow, membrane: grey and lumenal particles: red.

Movie S3. Tomographic slices of a hiPSC-derived neuronal process showing the ultrastructure of the MT cytoskeleton. Related to Figure 1E. Color coding, MT: yellow, membrane: grey and lumenal particles: red.

Movie S4. Tomographic slices of a Taxol-treated pluripotent P19 cellular process showing the ultrastructure of the cytoplasm containing MTs. Related to Figure S7C. Color coding, MT: yellow and lumenal particles: red.

Movie S5. Tomographic slices of a pluripotent P19 cellular process showing freshly polymerized MTs after Nocodazole treatment and washout. Related to Figure 6I. Color coding, MT: yellow and lumenal particles: red.

## Data Availability

Cryoelectron tomograms included in this manuscript and all maps generated in this work have been deposited in the EMDB, with the following accession numbers: Primary neuron tomogram, EMD-15474 (92); hiPSC-derived neuron tomogram, EMD-15437 (93); Pluripotent tomogram P19, EMD-15440 (94); Differentiated P19 tomogram, EMD-15438 (95); Nocodazole-treated P19 tomogram, EMD-15442 (96); Taxol-treated P19 tomogram, EMD-15443 (97); bMIP1, EMD-15453 (98); bMIP2, EMD-15454 (99); bMIP3, EMD-15455 (100); bMIP4, EMD-15456 (101); fMIP1, EMD-15441 (102); fMIP2, EMD-15439 (103); fMIP3, EMD-15436 (104); fMIP4, EMD-15444 (105); fMIP5, EMD-15457 (106); fMIP6, EMD-15458 (107); fMIP7, EMD-15459 (108); fMIP8, EMD-15472 (109); fMIP9, EMD-15460 (110); fMIP10, EMD-15461 (111); fMIP11, EMD-15462 (112); fMIP12, EMD-15463 (113); fMIP13, EMD-15464 (114); fMIP14, EMD-15466 (115); fMIP15, EMD-15467 (116); fMIP16, EMD-15468 (117); 80S ribosome average, EMD-15469 (118); primary neurons MT average, EMD-15470 (119). Proteomics data have been submitted to PRIDE with accession number PXD035700. Tomogram preprocessing (https://github.com/williamnwan/TOMOMAN) (120) and PySeg scripts (https://github.com/anmartinezs/pyseg_system) (46) are available on GitHub. All study data are included in the article and/or supporting information.
